# Influence of the Smile Line on Smile Attractiveness in Short and Long Face Individuals

**DOI:** 10.1155/2017/2637148

**Published:** 2017-08-08

**Authors:** Amjad Al Taki, Thar Hayder Mohammed, Ahmad Mohammad Hamdan

**Affiliations:** ^1^Smile Spa Dental Clinic, Dubai, UAE; ^2^Ajman University of Science and Technology, Ajman, UAE; ^3^Department of Orthodontic & Pediatric Dentistry, Faculty of Dentistry, University of Jordan, Amman, Jordan

## Abstract

**Objectives:**

The study assessed the impact of facial height on attractiveness of smile, in association with the maxillary gingival display. This research was performed by dental professionals and laypersons.

**Materials and Methods:**

Frontal extraoral photographs were captured for both short and long faces. The photographs were modified using software for image-processing and three rater groups (orthodontists, dentists, and laypersons) evaluated the smile attractiveness, with 30 subjects in each group. Differences in ratings of the different smiles among the different experimental groups were examined using the Kruskal-Wallis test. The Mann–Whitney* U* test was performed for pairwise comparisons between the experimental groups.

**Results:**

Dentists and laypeople were most likely to agree. For the short face, laypeople and dentists both rated the +2 mm gingival display smile as the most attractive smile whilst orthodontists ranked the 0 mm gingival display smile as the most attractive smile. For the long face, laypeople and dentists ranked the 0 mm gingival display smile as the most attractive smile, whilst orthodontists ranked the +2 mm gingival display as the most attractive.

**Conclusion:**

Smile line of both short and long face subjects was found to influence the smile attractiveness rating by the three rater groups.

## 1. Introduction

The interpretation of a smile is indicative of its nature, either pleasure, amusement, or derision. In other words, smile is a distinguished facial expression marked by an upward curve of the mouth at corners [1]. Also, it influences the perceived facial attractiveness of an individual and is used as an effective tool for social interaction. Previous study shows that the trust factor is higher for the people who smile as compared to nonsmiling people [2]. Smiles can be classified into two forms: (1) those of enjoyment, termed the Duchenne smile, and (2) the posed or social smile [3, 4]. The ability to smile has been learned by humans as part of evolution [5] and is important in the context of dentistry and orthodontics since there is repeatability of a posed smile over time [6, 7]. Preserving the correctness of a posed smile and ensuring flawlessness are important for a person and should never be ignored in diagnosis and treatment planning. A faultless smile helps maintain healthy relations through expression of friendliness, appreciation, and agreement and also conveys compassion and understanding [8].

In recent years, orthodontic diagnosis has progressed to include aesthetic diagnosis and treatment planning based on the patient's requirements, combined with the traditional problem-oriented approach. A previous study identified the key elements provided by the smile analysis report in the diagnosis and treatment planning. This has been made possible due to emergence of the soft tissue paradigm in the field of clinical orthodontics [9]. The smile display zone is determined by the thickness of the lips, structure of the gingival, smile index, intercommissural width, and the interlabial gap [10].

The perception of aesthetics varies individually based on the gender, age, and ethnicity, influenced by personal experiences and social environments [11]. For similar reasons, the opinions may differ with regard to the appreciation of beauty between the layman and professionals [12]. As shown by a previous study, a natural profile outline was preferred more by laypeople as compared to dentists [13]. Similarly, differences in smile appreciation have been reported between orthodontists and their patients [14], as well as between general dentists, orthodontists, and laypersons [14–16].

An understanding of these differences in opinion is important in ensuring that orthodontic treatment is planned to provide the best possible outcome for the patient. However, qualification and quantification of beauty are variable and therefore uncertain. The concepts regarding facial aesthetics are usually not based on proven scientific data, rather on subjective opinions. Hence, there is a need for sound scientific evidence in the orthodontic diagnosis and treatment planning despite the complexity [16]. However, the concerns of a patient regarding their facial aesthetics, especially their smile, are mostly influenced by their social surrounding as compared to their dental professionals.

Studies have shown that evaluation of aesthetics is affected by the key factors like social status, culture, and education level [17]. There might be differences in judging among the patients and orthodontists regarding the clinical result obtained after orthodontic treatment. This difference in perception makes it complicated to specify any satisfaction criteria related to orthodontic treatment [18, 19].

It has also been suggested that the macroaesthetics of a smile might depend on facial types [20] and certain variables that affect the smile aesthetics as well as attractiveness, which have been evaluated separately [16, 17, 19, 21]. Facial type (long face, normal face, and short face) may also be an important factor in aesthetics. A facial index provides clinicians with the general proportion of facial height (N to Gn) relative to facial width (Zy to Zy). The facial index for mesocephalic individuals is 90. Patients with a brachycephalic structure tend to have a shorter facial height relative to the width of the face. Patients with a dolichocephalic structure tend to have a longer, narrower face.

A study by Ackerman showed that the macroaesthetics of a smile might be dependent on the facial type. In support of the findings, a concomitant reduction in smile aesthetics could be an outcome for the patients with a brachycephalic face, which could exaggerate a transverse facial imbalance and produce the illusion of a flatter smile arc. On the contrary, in case of dolichocephalic patients, inadequate tooth mass in the buccal corridors or a wide buccal corridor could make the vertical facial imbalance more prominent and worsen the macroaesthetics of smiles.

There are few researches in support of frontal facial appraisal and the interactive influence of smile variables on aesthetics. This research gap contributed towards Hulsey's finding, exhibiting lower smile scores for the patients undergoing orthodontic treatment as compared to the patients with a normal occlusion, which was untreated [22]. Few studies have compared the effects of incisor display on smile aesthetics of patients possessing different facial types (short or long face). With diverging professional perception of smile attractiveness among the dental experts and the laypeople, the present study aims at quantitatively evaluating the influence of the smile line associated with the maxillary gingival display which was taken into consideration for both the short and long faced patients.

## 2. Materials and Methods

The study site included cities of Ajman, Sharjah, Ras Al Khaimah, and Dubai. The Ethics Committee of the Ajman University of Science and Technology approved the study. Written and informed consent was obtained from the participants, for publishing their images in the study report.

A total of 30 laypeople, 30 orthodontists, and 30 general practitioner dentists were engaged in the study. The mean ages of the three assessor groups were 40.07 ± 15.07 years, 36.00 ± 12.61 years, and 35.13 ± 8.84 years, respectively. The orthodontists and general practitioner dentists had an average experience of 10.20 ± 7.23 and 11.74 ± 6.56 years, respectively (Table 1).

### 2.1. Preparation of Smile Pictures

The study participants consisted of two patients, with a short and long face, respectively. The subjects were selected on a clinical basis and had vertical frontal measurements closely matching the values based on the subjective analysis of the face [23]. The frontal face of the patients was photographed in a standing and normal head position using a digital camera (Nikon D200, Tokyo, Japan). The clinically determined facial height values were remeasured and verified by evaluating printed version of the photographs. For long face, the facial height was considered as 137 mm and for short face it was 120 mm.

**Figure 1:**
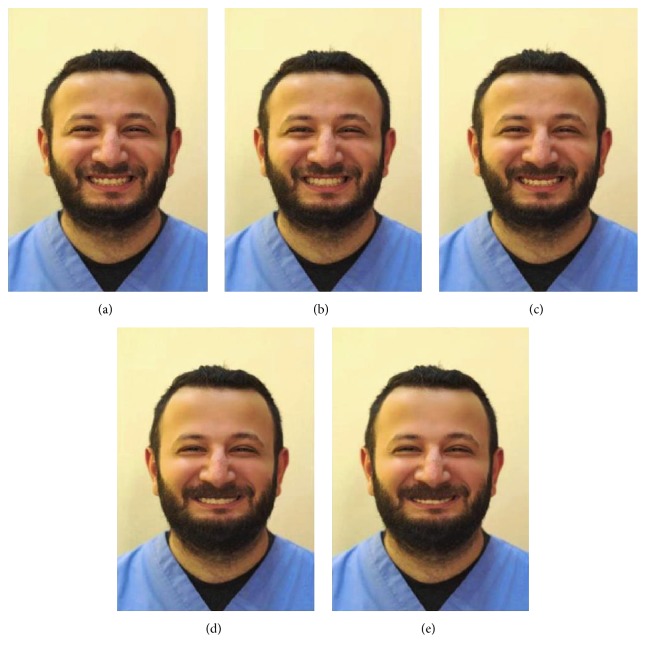
Short face smiles. (a) Short face +2 mm; (b) short face 0 mm; (c) short face +4 mm; (d) short face −2 mm; (e) short face −4 mm.

**Figure 2:**
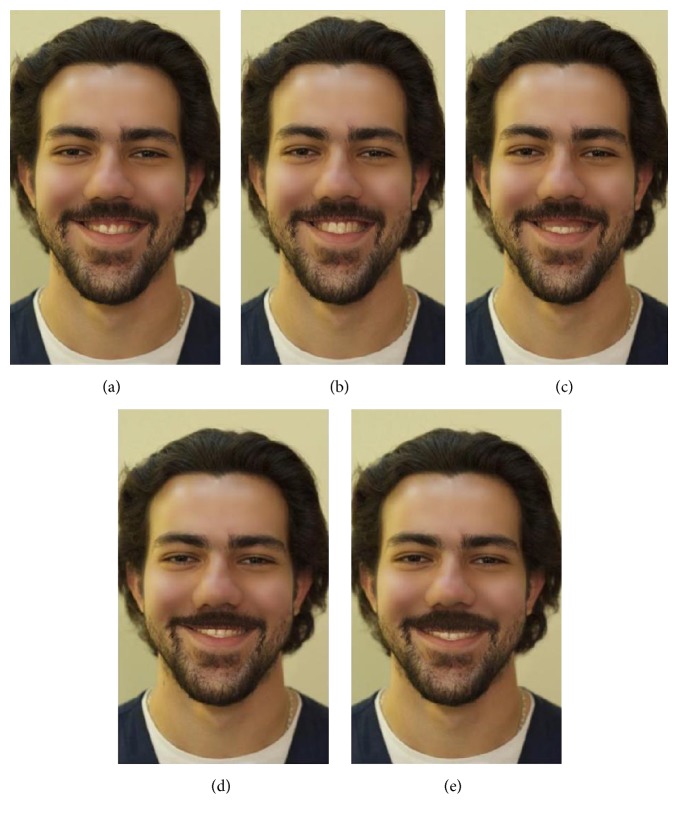
Long face smiles. (a) Long face +2 mm; (b) long face 0 mm; (c) long face +4 mm; (d) long face −2 mm; (e) long face −4 mm.

Slight imperfections (skin acne, scars) marked in the photograph was corrected by using Adobe Photoshop CS5 (Adobe Systems). The imperfections, if present, could have influenced the assessment of smile attractiveness and modification of smile into five different types according to gingival display (−4 mm, −2 mm, 0 mm, +2 mm, and +4 mm gingival display; Figures 1 and 2).

In addition, two booklets (for long and short face, resp.), each consisting of five randomly ordered frontal facial silhouettes (−4 mm, −2 mm, 0 mm, +2 mm, and +4 mm gingival display), were prepared. Each image was presented in a separate page with a 1–5 numerical rating scale, with 1 being the least attractive and 5 the most attractive.

### 2.2. Assessment of Smiles

To determine the variation of smile aesthetics with relation to the facial heights, a survey of 30 orthodontists, 30 general dentists, and 30 laypeople was conducted on the altered smile images of the long and short faced subjects. The majority of orthodontists and general dentists were males, with an average professional experience of more than 10 years. Available laypeople were contacted and those with dental affiliations were excluded. The majority of laypeople were males and college educated.

The raters were provided with instructions about the use of the questionnaire, including assurance that the rating was based on the overall smile and not related to other facial features. The booklet also included a 5-point, 1–5 numerical rating scale for each picture, with 1 being the least attractive and 5 the most attractive. The raters provided a score for each image. The booklets were presented to the assessors in random order.

### 2.3. Statistical Methods

The statistical analysis was done using the statistical software SPSS version 22.0 for Windows (SPSS, Chicago, IL, USA). In case of continuous variables, the demographic characteristics were presented as means and standard deviations (SD). The variation in the ratings of the different smiles between the different assessor groups was evaluated using the Kruskal-Wallis test. Pairwise comparisons between the groups were examined using Mann–Whitney* U* tests. Medians were compared using Independent Samples Median Tests. For all tests, the significance level was set at the 0.05.

## 3. Results

**Figure 3:**
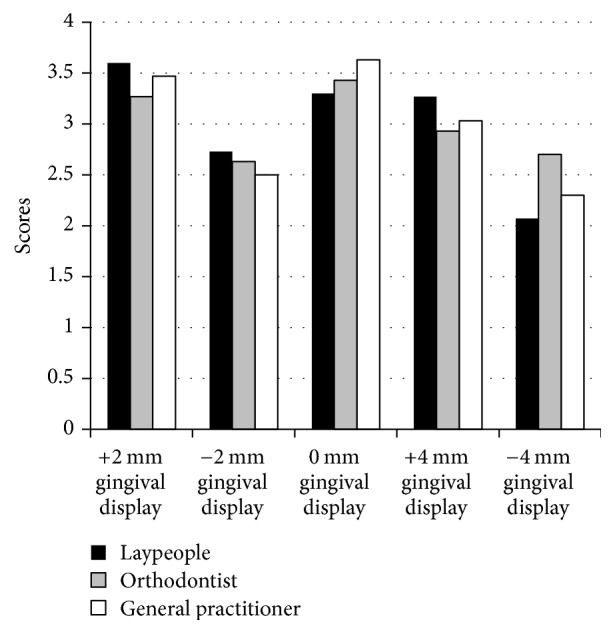
Graphic illustration of numerical rating scale means among groups for short faces.

**Figure 4:**
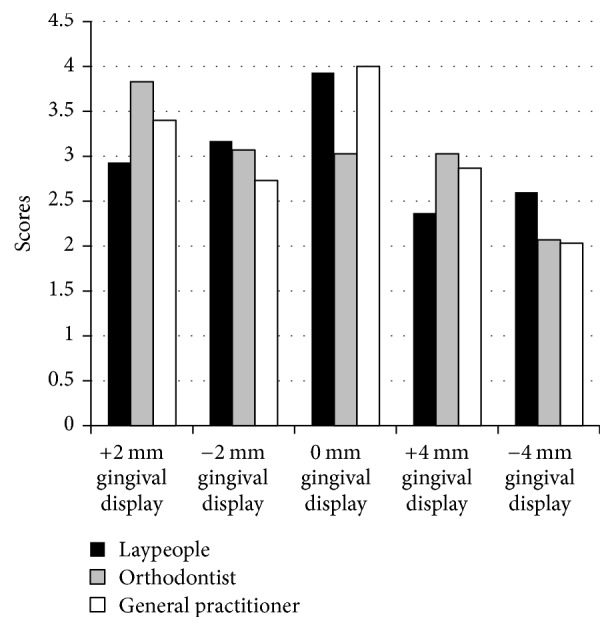
Graphic illustration of numerical rating scale means among groups for long faces.

Figures 3-4 and Tables 2-3 show the median scores for the different assessors and for each of the different smiles. There were large variations in perceived attractiveness between the different smiles. The laypeople and general practitioner dentists both rated the smile exhibiting +2 mm of gingival display in the short face subject as the most attractive. The smile showing +4 mm of gingival display ranked second. In contrast, orthodontists ranked the smile showing no gingival display as the most attractive in the short face. For the long face, laypeople and general practitioner dentists ranked the smile showing no gingival display as the most attractive, whilst orthodontists ranked a +2 mm gingival display as the most attractive.

Table 3 shows the median score for each of the 10 smiles. The distribution and medians across the three groups were assessed using Mann–Whitney* U* tests and Independent Samples Median Tests, respectively. For the majority of smiles, it was found that the distributions and the medians were the same in the 3 groups. The variation between the assessors was more apparent in the long face smiles, whereas the assessments of the short face smiles were similar across the three groups and no significant differences were identified. However, there was a significant difference in the distributions of long face subject displaying +2 mm of gingiva and for the long face subject exhibiting no gingival display. There were also significant median differences in the short face subject displaying −4 mm of gingiva and for the long face subject with no gingival display.

For the short face subject, the −4 mm smile was considered the most unattractive to the laypeople and the general practitioner dentists, followed by the −2 mm smile. The orthodontists ranked the −2 mm smile as the most unattractive, with the −4 mm smile as the second least attractive. In the long face subject, the orthodontists and general practitioner dentists ranked the −4 mm smile as the most unattractive. The lay group ranked the +4 mm smile as the most unattractive.

A pairwise analysis was conducted to determine differences between individual groups (Table 4). It was found that ratings for the short face smiling subjects were not significantly different across any of the pairings. However, for the long face smiling subjects, significant differences in the ratings of +2 mm and 0 mm gingival display between the laypeople and the orthodontists were found. A significant difference between general practitioner dentists and orthodontists in the rating of the 0 mm gingival display was found.

## 4. Discussion

Smile aesthetics are a primary cause for seeking orthodontic treatment for patients with dental problems as well as for orthodontists. The results indicated that laypeople and general practitioner dentists largely agreed in their assessments of smile aesthetics, whilst there were some differences in the assessments determined by orthodontists. Overall, the extreme gingival display smiles were not rated favorably by any of the assessment groups.

The first investigation of the variables that might contribute to aesthetic smiles was an innovative study by Kokich et al. [15] who used amended photographs with only visible lips and teeth for fabrication of 5 image variations with 8 characteristics. The participants were requested to judge the attractiveness of the smile in the modified photographs on a visual analogue scale (VAS). The results showed that the orthodontists, laypeople, and dentists were able to detect changes in smile characteristics at different threshold levels. The most forgiving was the laypeople group that defined patient values based on smile characteristics. Brisman [24] extended this work by comparing photographs as well as drawings of maxillary central incisors of differing symmetry, shape, and proportion. A survey on dental students, dentists, and laypeople showed significantly varying preferences among each group.

Several researchers have performed studies on the frontal facial form, to determine the characteristics that are aesthetically desirable. The symmetry of teeth along the lip curvature and the amount of gingival display have significant effects on smile aesthetics [19, 22, 25, 26]. This supports the findings of the present study, which demonstrated large differences in the attractiveness rating depending on the level of gingival display. Notably, the extreme positive or extreme negative (±4 mm) gingival displays were rated as the most unattractive of the smile types in both long and short faces and by all three assessor groups.

In the present study, there were similarities in the way that participants assessed and rated the different smile processes. Laypeople and general practitioner dentists were the most closely matched of the three assessor groups, with no significant differences identified in their rating of any of the smiles. In contrast, differences in the assessments provided by the orthodontists and those provided by the laypeople and general practitioner dentists were observed, which showed the acceptance of the full height of the maxillary incisors plus 1 to 2 mm of gingiva by the orthodontists that needs to be displayed during smiling. This knowledge increases orthodontic tolerance of gingival display, unlike people from other professions. The present findings agree with those of Al Taki and Guidoum [27], who observed similar perceptions in facial aesthetic appearance between laypeople, dental students, general practitioners, oral surgeons, and orthodontists. In contrast, Zange et al. [28] found that laypeople were more critical of aesthetics than orthodontists.

There are some potential limitations to this study. The mean age of the assessors was higher than the ages of the two faces assessed. It is unknown whether age of assessor compared to age of subject may impact on smile perception and further work may assess this. In our study we used just two faces and systematically altered the facial height to assess smile perception. It remains unclear whether this is the most appropriate method for assessing smile perception or whether studies should include multiple faces in each cephalometric category. Both of the faces in our study had facial hair. The impact of facial hair on smile perception is unclear and further work may include faces both with and without facial hair to provide comparison.

Additional areas on which further work should focus include other characteristics which may influence the perception of smile attractiveness between people of different social, educational, and demographic backgrounds in order to assist orthodontists in developing the most appropriate treatment plan.

## 5. Conclusions

- The maxillary gingival display associated smile line in both the short and long faced subjects influences attractiveness perception by laypeople, orthodontists, and general practitioner dentists.
- There were similarities in the way that the three assessor groups rated the different smiles, particularly for the short faces. The differences were more pronounced in long faced subjects, which suggested that an interpretation of smile attractiveness may need different levels of consideration during the development of treatment plans for patients with varying facial types.

## Figures and Tables

**Table 1:** Demographics of scorers.

	Laypeople *n* = 30	Orthodontists *n* = 30	General practitioner dentists *n* = 30
Age (yrs), mean ± SD	45.07 ± 15.07	36.00 ± 12.61	35.13 ± 8.84
Experience (yrs), mean ± SD	—	10.20 ± 7.23	11.74 ± 6.56

**Table 2:** Comparison of median scores given by laypeople, orthodontists, and general practitioner dentists for short faces.

Short face					
	+2 mm gingival display	−2 mm gingival display	0 mm gingival display	+4 mm gingival display	−4 mm gingival display
Laypeople, median	4 (3, 5)^ †^	3 (2, 4)	3 (2, 4)	3.5 (2, 5)	2 (1, 2.25)
Orthodontist, median	3 (2, 5)	2 (2, 4)	4 (2, 5)	3 (1, 5)	3 (1, 4)
General practitioner, median	3 (2, 5)	2 (1, 3.25)	4 (3, 4)	3 (1.75, 5)	2 (1, 3.25)
*P* (median)^*∗*^	0.429	0.732	0.434	0.730	**0.031**

^*∗*^Independent Samples Median Test. ^†^Median (in quartiles).

**Table 3:** Comparison of median scores given by laypeople, orthodontists, and general practitioner dentists for short faces.

Long face					
	+2 mm gingival display	−2 mm gingival display	0 mm gingival display	+4 mm gingival display	−4 mm gingival display
Laypeople, median	3 (1.75, 4)^†^	3 (2, 4)	4 (3, 5)	2 (1, 3.25)	2 (1, 4)
Orthodontist, median	4 (3, 5)	3 (2, 4)	3 (2, 4)	3 (2, 4.25)	2 (1, 3)
General practitioner, median	3 (2.75, 4.25)	3 (1, 4)	4 (3, 5)	3 (1.75, 4)	2 (1, 3)
*P* (median)	0.057	0.574	0.029^**∗**^	0.113	0.218

^*∗*^Independent Samples Median Test. ^†^Median (in quartiles).

**Table 4:** Pairwise comparisons (Mann–Whitney *U* test) between the three groups of scorers for the 10 smile types.

	Lay people versus orthodontist	Laypeople versus general practitioner	Orthodontist versus general practitioner
Short face			
+2 mm gingival display	0.296	0.577	0.588
−2 mm gingival display	0.770	0.378	0.599
0 mm gingival display	0.601	0.322	0.830
+4 mm gingival display	0.423	0.576	0.791
−4 mm gingival display	0.069	0.537	0.260
Long face			
+2 mm gingival display	**0.007**	0.205	0.117
−4 mm gingival display	0.762	0.215	0.333
0 mm gingival display	**0.006**	0.795	**0.004**
−2 mm gingival display	0.083	0.167	0.656
+4 mm gingival display	0.141	0.132	0.956
